# Recent Advances in TiO_2_-Based Photocatalysts for Reduction of CO_2_ to Fuels

**DOI:** 10.3390/nano10020337

**Published:** 2020-02-17

**Authors:** Thang Phan Nguyen, Dang Le Tri Nguyen, Van-Huy Nguyen, Thu-Ha Le, Dai-Viet N. Vo, Quang Thang Trinh, Sa-Rang Bae, Sang Youn Chae, Soo Young Kim, Quyet Van Le

**Affiliations:** 1 Laboratory of Advanced Materials Chemistry, Advanced Institute of Materials Science, Ton Duc Thang University, Ho Chi Minh City 700000, Vietnam; nguyenphanthang@tdtu.edu.vn; 2 Faculty of Applied Sciences, Ton Duc Thang University, Ho Chi Minh City 700000, Vietnam; 3 Institute of Research and Development, Duy Tan University, Da Nang 550000, Vietnam; dltnguyen@yahoo.com; 4 Key Laboratory of Advanced Materials for Energy and Environmental Applications, Lac Hong University, Bien Hoa 810000, Vietnam; vhnguyen@lhu.edu.vn; 5 Faculty of Materials Technology, Ho Chi Minh City University of Technology (HCMUT), Vietnam National University–Ho Chi Minh City (VNU–HCM), 268 Ly Thuong Kiet, District 10, Ho Chi Minh City 700000, Vietnam; lthuha2410@gmail.com; 6 Center of Excellence for Green Energy and Environmental Nanomaterials (CE@GrEEN), Nguyen Tat Thanh University, 300A Nguyen Tat Thanh, District 4, Ho Chi Minh City 755414, Vietnam; daivietvnn@yahoo.com; 7 Cambridge Centre for Advanced Research and Education in Singapore (CARES), Campus for Research Excellence and Technological Enterprise (CREATE), 1 Create Way, Singapore 138602, Singapore; qttrinh@ntu.edu.sg; 8 Department of Materials Science and Engineering, Korea University, 145 Anam-ro, Seongbuk-gu, Seoul 02841, Korea; dngorl147@naver.com; 9 Department of Materials Science, Institute for Surface Science and Corrosion, University of Erlangen-Nuremberg, Martensstrasse 7, 91058 Erlangen, Germany

**Keywords:** photocatalysis, carbon dioxide reduction, TiO_2_-based photocatalysts, high efficiency

## Abstract

Titanium dioxide (TiO_2_) has attracted increasing attention as a candidate for the photocatalytic reduction of carbon dioxide (CO_2_) to convert anthropogenic CO_2_ gas into fuels combined with storage of intermittent and renewable solar energy in forms of chemical bonds for closing the carbon cycle. However, pristine TiO_2_ possesses a large band gap (3.2 eV), fast recombination of electrons and holes, and low selectivity for the photoreduction of CO_2_. Recently, considerable progress has been made in the improvement of the performance of TiO_2_ photocatalysts for CO_2_ reduction. In this review, we first discuss the fundamentals of and challenges in CO_2_ photoreduction on TiO_2_-based catalysts. Next, the recently emerging progress and advances in TiO_2_ nanostructured and hybrid materials for overcoming the mentioned obstacles to achieve high light-harvesting capability, improved adsorption and activation of CO_2_, excellent photocatalytic activity, the ability to impede the recombination of electrons-holes pairs, and efficient suppression of hydrogen evolution are discussed. In addition, approaches and strategies for improvements in TiO_2_-based photocatalysts and their working mechanisms are thoroughly summarized and analyzed. Lastly, the current challenges and prospects of CO_2_ photocatalytic reactions on TiO_2_-based catalysts are also presented.

## 1. Introduction

Heavy dependence on fossil fuels since the past few centuries for industrialization and economic growth has depleted the sources of carbon-emitting fossil fuels and has led to record-breaking atmospheric concentrations of carbon dioxide (CO_2_), which is a major component of greenhouse gases, thus contributing to global warming and climatic changes [1,2,3]. To mitigate the problematic impacts of CO_2_ on human life and the environment, efforts have been made to reduce carbon emissions caused by capturing, storing, and transforming CO_2_ into other valuable substances [4,5,6]. In addition, CO_2_ is considered as one of the cheapest and most abundant carbon resources to produce various C_1_ products such as carbon monoxide (CO), methane (CH_4_), formic acid (HCOOH), and methanol (CH_3_OH), which are critical for the synthesis of other value-added chemicals [7,8].

Lately, the electroreduction of CO_2_ and photoreduction strategies have been paid increasing attention to as promising procedures to utilize CO_2_ as a carbon building-block for synthesizing hydrocarbon fuels [9,10,11]. Electroreduction of CO_2_ requires electricity as the driving force for the electrochemical reaction [12,13,14,15]. Therefore, the requirement of the energy input is concerning. This energy can be generated from non-renewable energy resources. Meanwhile, solar energy is among the most critical renewable energy sources along with wind energy, wave energy, and hydrogen energy [16]. Photoreduction systems activated by solar energy are a green but intermittent power source for catalyzing the reduction of CO_2_. Such systems have been considered one of the most sustainable and cost-effective approaches to exploit solar energy combined with the utilization of anthropogenic CO_2_ as a starting material of the carbon cycle to store energy in the form of chemical fuels [8,17,18].

Titanium dioxide (TiO_2_) has been the benchmark for photocatalytic studies over nearly four decades since the seminal work by Fujishima and Honda, who showed the high photochemical stability, low-cost features, facile preparation, abundance, and low toxicity of TiO_2_ [19]. TiO_2_ exhibits the most extensive applications as a semiconductor and can be used in a wide range of photocatalysis applications including water splitting, photodegradation of pollutants, dye bleaching, and water desalination, thus contributing to solving the environmental and energy crisis [20,21,22,23,24,25,26,27]. Parallel to water splitting technologies, an extensive line of studies on TiO_2_-based photocatalysts for CO_2_ reduction has been conducted to overcome obstacles including limited light-harvesting, charge separation hindering improved process efficiencies, inefficient selectivity of products, the inability to suppress the competing the hydrogen evolution reaction (HER), and catalytic instability [28,29,30,31]. In this study, we therefore review the photocatalytic conversion of CO_2_ to fuels using the recently advanced TiO_2_-based materials. The fundamentals of CO_2_ photoreduction are discussed to understand the factors affecting the performance of photocatalysts, and the updated progress in the enhancement of its efficiency is comprehensively discussed. The effective strategies for overcoming the limitations of TiO_2_-based photocatalysts are categorized as follows: (i) Design of nanostructured TiO_2_-based catalysts, (ii) modification of TiO_2_ facial properties, and (iii) cocatalysts for TiO_2_ photocatalysts. Lastly, the current challenges and directions for future progress are provided, which could offer guidelines for the future development of CO_2_ reduction on TiO_2_-based photocatalysts.

## 2. Fundamentals of CO_2_ Photocatalytic Reduction

Thermodynamically, CO_2_ is one of the most stable linear molecules, showing the highest oxidation state of the carbon atom with a Gibbs free energy of ∆G^o^ = −394.4 kJ mol^−1^. A high input energy is required to activate the CO_2_ molecule, following the equation:

∆G^o^ = nFE^o^. (1)

In reality, however, to break the C=O bond and bend the linear geometry of CO_2_, a more negative potential than E^o^ is required to make the ∆G^o^ value negative enough to spontaneously accelerate the conversion of CO_2_ and to compensate for the overpotential (Equation (2)):

E = η + E^o^ (2)

where ∆G^o^ is the standard Gibbs free energy, E is the required potential, E^o^ is the standard potential, η is the overpotential, n is the number of transferred electrons, and F is the Faraday constant.

In addition, the presence of reductants providing protons to react with the adsorbed CO_2_ on a photocatalyst surface and electrons to form products assists the process of CO_2_ conversion by lowering the required input energy in comparison to the absence of reducing agents, in which the reduction of CO_2_ proceeds by a photocatalyst alone and is much more difficult because of the high stability of inert CO_2_. Therefore, photoreduction of CO_2_ is commonly conducted with a reductant, such as H_2_ or/and H_2_O, making the conversion of CO_2_ into fuels more feasible and more efficient from the view point of consumed energy [32,33]. In a CO_2_(g)/H_2_O(g/L) or CO_2_(g)/H_2_(g) system, photoexcited electrons are generated under light irradiation and are transferred, and then they react with the adsorbed CO_2_ on the photocatalyst surface along with protons provided by the reducing agents to yield the reduced products. H_2_O (vapor or liquid) is well known as an inexpensive and safer reductant than H_2_ gas which is dangerous, although HER is also coupled in the H_2_O phase, which lowers the CO_2_ reduction efficiency because of their similar potentials for generating intermediates. Various products from the CO_2_ reduction reaction combined with the competing HER and their standard potentials are presented in Table 1.

Importantly, the formation of unstable radical anion CO_2_^−^ has been proved to be the most widely recognized mechanism to activate CO_2_ molecules in the initial stage of the photoreduction process. However, this activation requires a very negative equilibrium potential of −1.49 V vs. reversible hydrogen electrode (RHE) at pH = 7 to form the CO_2_^−^ intermediate, resulting in a significant increase in the required negative potential applied for electrochemical reduction of CO_2_ compared with the standard potentials in Table 1.

**Figure 1:**
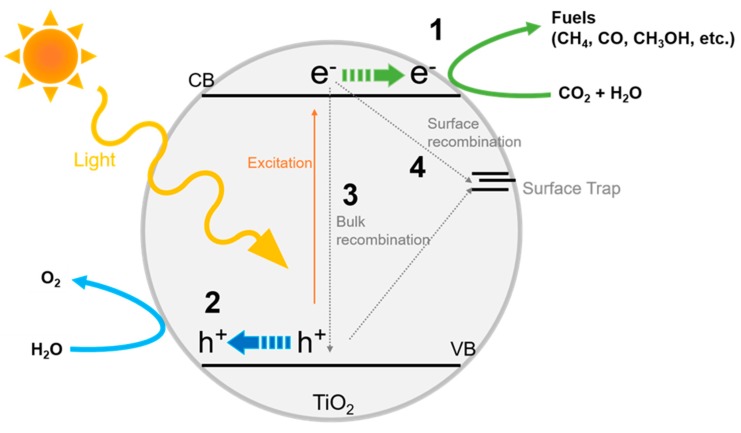
Schematic illustration of photoreduction of CO_2_ into fuels on TiO_2_-based photocatalysts.

The overall principles of the photoreduction CO_2_/H_2_O(g/L) system are illustrated in Figure 1. Under light irradiation, incident photons with an equal or higher energy than that of the bandgap semiconductor (e.g., 3.2 eV for anatase TiO_2_), which act as the photocatalyst, are absorbed and the pair-charges, electrons and holes, are generated. This is followed by the separate transportation of the generated electrons and holes to the photocatalyst surface. Subsequently, the photogenerated electrons reduce CO_2_ to value-added chemicals (step number 1) while water is oxidized by holes to oxygen molecules (step number 2). The recombination of the electron-hole pairs, which may consume a lot amount of photogenerated charges, in the bulk and on the surface can occur in step numbers 3 and 4, respectively. Proton reduction, which is called HER, appears to be spontaneous in step number 1 under aqueous conditions and competes with the reduction of CO_2_.

**Figure 2:**
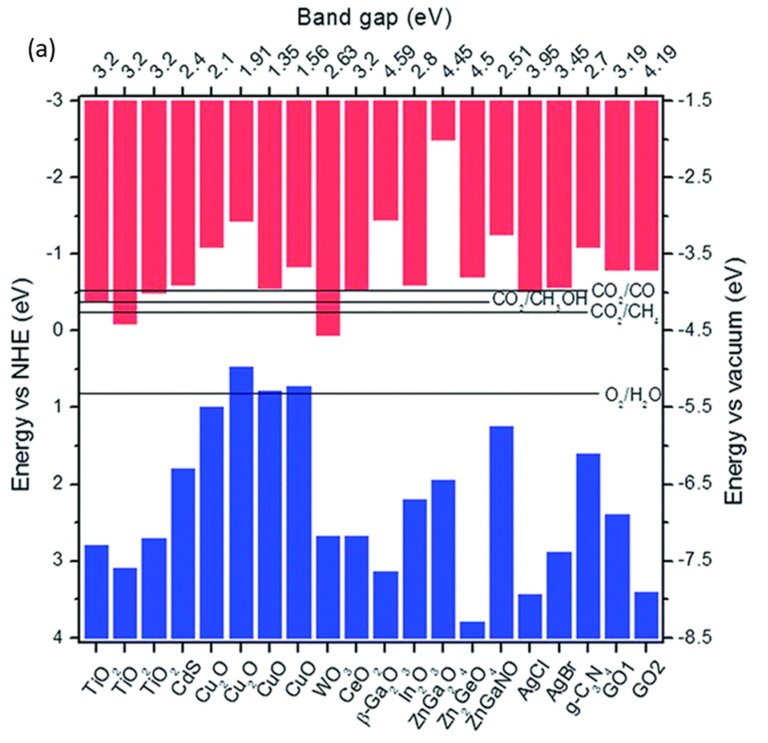

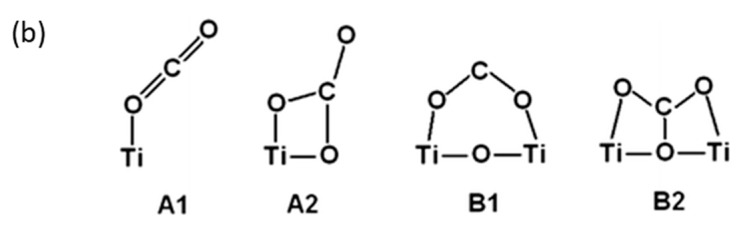
(**a**) conductive band (CB), valence band (VB), and bandgap energies of several semiconductors summarized from the references [34,35,36,37,38,39,40]. Reproduced with permission from [34]. Copyright 2015, Royal Society of Chemistry. (**b**) Configurations of CO_2_ molecule on TiO_2_ surface. Reproduced with permission from [41]. Copyright 2012, American Chemical Society.

For the selection of the photocatalyst material, the conductive band (CB) and valence band (VB) positions of semiconductors are critical factors, which help analyze the possibility for the redox reaction. Alternative semiconductors possess different CB and VB positions, thus varying the band gap, which is the potential energy between the CB and VB (Figure 2a) [34,35,36,37,38,39,40]. Theoretically, for instance, anatase TiO_2_ with CB and VB potentials of −0.5 eV and 2.7 eV, respectively, enables the reduction of CO_2_ into several fuels (such as CH_4_, CH_3_OH, and CO) and oxidizes H_2_O because its CB is more negative than the reduction potential to form products, whereas its VB more positive than the redox potential of the reductant. Nonetheless, the high band gap (3.2 eV) requires a high energy of photons with a short wavelength (UV light) to activate photoexcitation.

Insights into the interaction of adsorbed CO_2_ with the semiconductor surface are important because the structure and composition of the catalytic sites can significantly vary the pathways and selectivity of different products. Thus, Curtiss et al. proposed diverse binding configurations of activated CO_2_ molecules on the TiO_2_ surfaces (Figure 2b) [41]. They also found that the cation sites on the Ti surface can lower the reaction barrier to activate CO_2_ and the stability of CO_2_^−^ intermediates to enhance CO_2_ photoreduction.

Briefly, the photoreduction of CO_2_ on TiO_2_ can encounter some intrinsic challenges such as the low light-harvesting efficiency, charge recombination, thermodynamic difficulty in activation of CO_2_, the required high negative overpotential for intermediates, the wide distribution of products from CO_2_ reduction in kinetics, and the competition of the favorable HER from H_2_O reduction. These limitations could be overcome to enhance the photocatalytic activity of CO_2_ on TiO_2_-based photocatalysts by focusing on some critical factors including surface molecular structures and charge transfer behaviors, which will be discussed in the following sections.

## 3. Advances in TiO_2_-Based Photocatalysts for CO_2_ Reduction

### 3.1. Nanostructured TiO_2_ Design

Various nanostructured TiO_2_-based photocatalysts with different surface molecular structures play a significant role on specifying the surface adsorption properties and surface electronic structures, and thus, the photocatalytic activity of a photocatalyst is determined by its electronic structure resulted from the light absorption efficiency and redox potential of the excited charges. In addition, the surface charge transfer configurations also contribute to the photocatalytic activity by accelerating electron and hole transportation and impeding electron-hole recombination [42,43]. To that end, many studies have reported advances for CO_2_ photoreduction on engineering of crystal phases [44,45,46,47,48], facets [41,49,50,51,52,53,54,55,56,57], and TiO_2_-dispersed on porous materials [58,59,60,61].

#### 3.1.1. Crystal Phases

**Figure 3:**
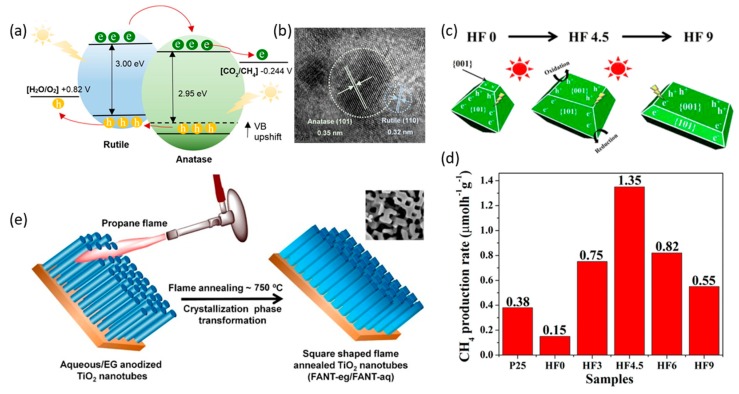
(**a**) Heterojunctions induced from rutile and anatase crystal phases of TiO_2_. (**b**) TEM images of anatase {101} and rutile phases {110}. (**a**,**b**) Reproduced with permission [47]. Copyright 2016, Royal Society of Chemistry. (**c**) Schematic illustration of the spatial separation of redox sites on TiO_2_ prepared by alternating HF amounts. (**d**) Comparison of the CH_4_ production rate of P25 and the TiO_2_ samples prepared by varying HF amounts. (**c**,**d**) Reproduced with permission [52]. Copyright 2014, American Chemical Society. (**e**) Transformation of the anatase phase of TiO_2_ nanotubes into the rutile phase with {110} as the dominant plane using the flame annealing method. (**e**) Reproduced with permission [56]. Copyright 2019, Elsevier.

TiO_2_ possesses three types of crystal phases: Anatase, rutile, and brookite. The first two crystal structures are usually used as photocatalysts. The photocatalytic activity can be improved by creating a mixture of different crystal structures rather than using a single crystal phase due to the improved charge separation and transfer efficiency induced by heterojunction effects [44,45,46]. To this end, Chai et al. controlled the composition of anatase and rutile phases in TiO_2_ to enhance the photoactivity by simply varying the annealing temperature [47]. As expected, TiO_2_ nanoparticles (NPs) were composed of both anatase and rutile crystal phases, leading to an increase in the electron-hole separation caused by the created heterojunction (Figure 3a,b). They also found that the optimal fraction of phase content for their TiO_2_ photocatalyst was 17.5% rutile phase. Moreover, another factor that contributes to the catalytic activity is morphology. A synergistic influence of crystal phase content and morphology can be expected to improve photocatalytic performance. To elucidate this hypothesis, 1-D hierarchical mesoporous TiO_2_ nanofibers were synthesized using combined electrospinning and sol-gel methods. The obtained photocatalyst exhibited higher activity than TiO_2_ NPs prepared only by the sol-gel procedure [48]. The fast charge transport and impeded electron-hole recombination caused by the nanofiber morphology led to improved activity. Meanwhile, the 1-D fiber photocatalyst with a higher ratio of rutile to anatase phases (20:80) produced four and 2.5 times higher H_2_ and CO, respectively, than the counterpart containing a lower ratio of rutile to anatase phase (7:93), revealing the crucial role of crystalline phase composition in photocatalytic activity.

#### 3.1.2. Effect of Facets

Facet engineering of TiO_2_ photocatalysts, which is another promising strategy to achieve high photocatalytic activity via CO_2_ adsorption on the catalyst surface, photoinduced activation, and charge separation, has been extensively studied [41,49,50,51,52,53,54,55]. In general, the {101} facet is more thermodynamically stable than {001} facets, suggesting that anatase TiO_2_ crystals usually possess the dominant exposed {101} facets [49]. Using first-principle calculations, Curtiss et al. investigated the binding configuration of intermediates on anatase {101} surfaces and found that the reduced {101} anatase surfaces favor CO_2_ binding along with charge transfer to CO_2_ [41,50]. As a result, the adsorption of CO_2_ and the charge transfer from the surface of anatase TiO_2_ photocatalysts to CO_2_ was theoretically demonstrated to be enhanced on the preferentially exposed {101} facets. Nevertheless, Han et al. unveiled that {001} facets are more reactive in photocatalytic reduction of CO_2_ than the low-energy {101} surfaces because of the high density of active unsaturated Ti atoms and active surface oxygen atoms [51]. Therefore, the coexposed facet approach is believed to promote spatial charge separation and form a facet-related heterojunction, thus resulting in longer charge carrier lifetimes, which can hinder electron-hole recombination and accelerate electron and hole transportation while maintaining the high adsorption of CO_2_. In a subsequent study, Yu et al. appropriately manipulated the efficient ratio of coexposed {101} and {001} facets to synergistically leverage the CO_2_ adsorption and reactivity on both facets [52]. By varying the HF amount used during the synthesis of TiO_2_ nanosheets, the growth of the {101} and {001} facets can be adjusted. Therein, the optimal HF amount used is 4.5 mL to obtain the {001} and {101} exposed facets in a proportion of 55:45, with a CH_4_ yield from photoreduction of CO_2_ of 1.35 μmol g^−1^ h^−1^ (Figure 3c,d).

Nonetheless, Kar et al. very recently reported an efficient method to transform anatase phase TiO_2_ nanotubes into the rutile phase with the {110} facet as the dominant plane using the flame annealing method (Figure 3e) [56]. Flame-annealed nanotubes prepared in a water-based electrolyte (FANT-aq) exhibited superior yield (156.5 μmol g^−1^ h^−1^) in the presence of the rutile phase as the only crystalline phase. High activity of the FANT-aq can be attributed to the increased visible-light absorption due to the reduced band gap induced by the pure rutile phase, defects and sub-energy levels, square morphology, and the preferentially oriented rutile {110} facet.

**Figure 4:**
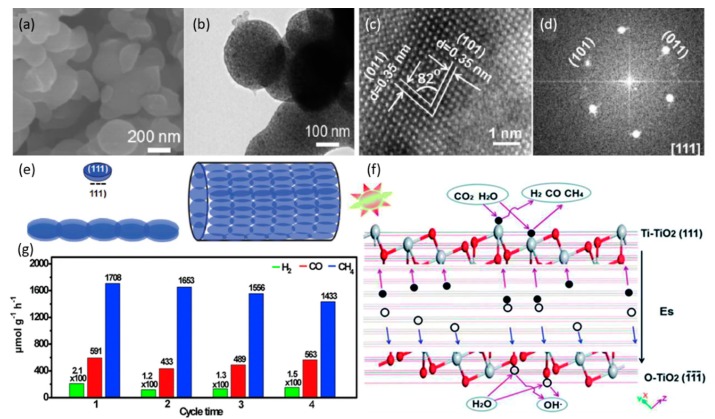
(**a**) SEM and (**b**) TEM images of the catalyst prepared in the TOBT-CH_3_COOH-H_2_O-H_2_SO_4_ system. (**c**) HRTEM image and (**d**) the corresponding FFT pattern from the box in (**b**). (**e**) Structural illustration of the TiO_2_ nanotubes/nanowires constructed with nanoflakes with exposed {111} crystal facets. (**f**) The schematic illustrations of charge separation under Es and photocatalytic reaction. (**g**) Photocatalytic CO_2_ reduction activity and stability in recycling the photocatalyst. (**a**–**g**) Reproduced with permission [57]. Copyright 2019, Royal Society of Chemistry.

Remarkably, Yang et al. lately prepared hydrogenated nanotube/nanowire photocatalysts assembled from TiO_2_ nanoflakes with an exposed clean {111} facet, which is believed to be the origin of the enhanced solar absorption and the good charge separation to attain a superior photocatalytic performance up to 1708.1 μmol g^−1^ h^−1^ CH_4_ at 373 K under the irradiation of a 300-W Xe lamp, which is one of the best performances for methane production. Importantly, a spontaneous electric field (Es) is demonstrated to exist between the polar TiO_2_ {111} and $\left(\bar{1}\bar{1}\bar{1}\right)$ planes, under which photoinduced electrons and holes migrate to the positive Ti–TiO_2_ {111} and negative O–TiO_2_ $\left(\bar{1}\bar{1}\bar{1}\right)$ polar surfaces, respectively, which is followed by the subsequent redox reactions to reduce CO_2_ into CH_4_, as shown in Figure 4 [57].

#### 3.1.3. Well-Dispersed TiO_2_ on Porous Materials

Porous materials have been utilized as a supporter for catalysts because of their high surface area, well-dispersed active sites, large adsorption capacities, and alternative pore sizes suitable for facile diffusion and transportation of gas and liquid reactants. Therefore, porous materials including zeolite and hierarchical silica have been considered as potential supporting candidates in photocatalytic reduction of CO_2_. As one of the earliest investigations, in a study by Anpo et al., highly dispersed TiO_2_ on zeolites (Ti-MCM) were synthesized by a hydrothermal method for photoreduction of CO_2_ to CH_4_ and CH_3_OH [58,59,60]. The well-dispersed Ti zeolites catalyzed the photoreduction of CO_2_ into CH_4_ and exhibited approximately 10-fold higher photocatalytic efficiency than TiO_2_ powder. Interestingly, high activity for CH_3_OH formation was achieved on Ti-MCM-48, whereas the bulk TiO_2_ counterpart only produced CH_4_ as the product. The results indicate that the 3-D channel structure of zeolites in Ti-MCM-48 is not only beneficial for the high dispersion of TiO_2_, but is also beneficial for the high amount of adsorbed CO_2_ and production of CH_3_OH. It is plausible that the formation of charge transfers excited complexes (Ti^3+^-O^−^)* on the isolated TiO_2_ species in zeolites. Moreover, OH∙ radicals are formed from the decomposition of H_2_O, which react with methyl from reduction of CO_2_ and finally generate CH_3_OH [59]. In a subsequent study, Ti-SBA-15 synthesized with a higher pore size exhibited 106 μmol CH_4_ g^−1^ h^−1^ production via photoreduction of CO_2_ into CH_4_ and CH_3_OH under UV irradiation. This activity is 20 times higher than that of Ti-MCM-41 and Ti-MCM-48 [61].

### 3.2. Modification on TiO_2_ Surface

Surface modifications of the crystal structure can act as active sites on metals and/or metal-oxide catalysts, modifying the electronic structure and binding strength of intermediates, and thus, influencing the catalytic activity. Two popular approaches on TiO2-based photocatalyst surface are related to oxygen vacancies [62,63,64,65,66,67,68,69,70,71,72] and basic-site functionalized modifications [73,74,75,76,77,78].

#### 3.2.1. Oxygen Vacancies

**Figure 5:**
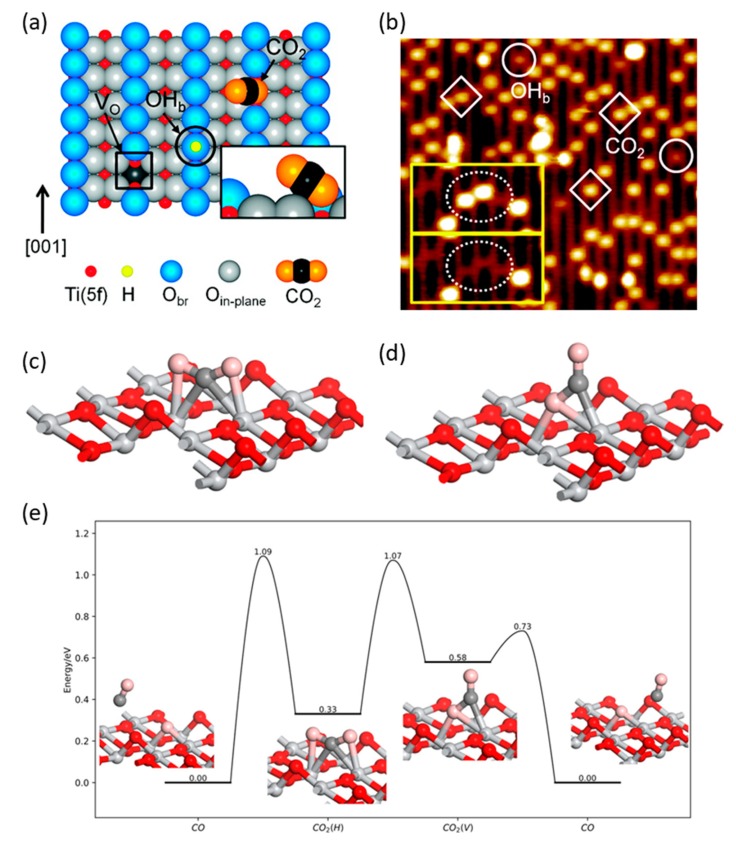
(**a**) Schematic representation showing V_o_, OH_b_, and CO_2_ molecule adsorbed at a V_o_ site on reduced TiO_2_. (**b**) Scanning Tunneling Microscope (STM) image of the TiO_2_ {110} surface after adsorption of CO_2_ at 55 K. (**a**,**b**) Reproduced with permission from [64]. Copyright 2011, American Chemical Society. (**c**–**e**) Configurations of adsorbed CO_2_ on O_v_ site: (**c**) CO_2_(H); (**d**) CO_2_(V); (**e**): Adsorption and dissociation of CO_2_ on the oxygen vacancy site. The Ti, O, and O in CO_2_ and C are shown in silver, red, pink, and gray colors, respectively. (**c**–**e**) Reproduced with permission from [65]. Copyright 2019, American Chemical Society.

Oxygen defects are one of the most famous and efficient approaches to enhance photocatalytic activities [62,63]. The behavior of CO_2_ adsorbed on oxygen defects (V_o_) on the TiO_2_ {110} surface was investigated by Lee et al. [64] Their results unveiled that with the existence of V_o_, one oxygen atom of CO_2_ can be situated at the position of the oxygen defect and can the CO_2_ molecule on TiO_2_ with a bridge formed by the adsorption of the oxygen atom from CO_2_, which can enhance CO_2_ trapping and lower the activation barrier (Figure 5a,b). Recently, Alexandrova et al. employed density functional theory calculations to systematically study the role of oxygen vacancies on anatase TiO_2_ {101} surface (Figure 5c–e) [65].

**Figure 6:**
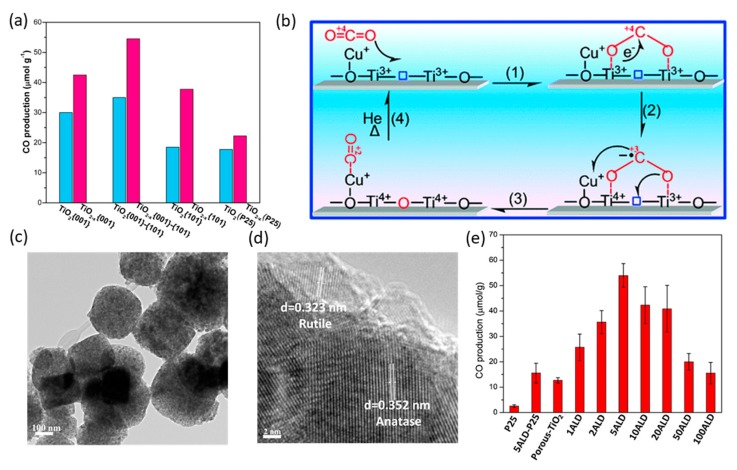
(**a**) Generation of CO on oxygen-deficient blue TiO_2_ nanocrystals with coexposed {101}-{001} facets in comparison with some other photocatalysts. Reproduced with permission from [66]. Copyright 2016, American Chemical Society. (**b**) A rational mechanism for spontaneous dissociation of CO_2_ on a defective Cu(I)/TiO_2−x_ catalyst even in the dark. Reproduced with permission from [68]. Copyright 2012, American Chemical Society. (**c**,**d**) TEM images of deposition of five atomic layers of MgO on porous TiO_2_. (**e**) CO production rate of different atomic layer deposited MgO surface coating layers on porous TiO_2_. (**c**–**e**) Reproduced with permission from [76]. Copyright 2018, Elsevier.

The significance of oxygen vacancies on defected TiO_2_ has been ascertained in the enhancement of CO_2_ adsorption, activation, dissolution, and stabilization of reaction intermediates. Li et al. found that the photocatalytic activity of the enriched oxygen defect on TiO_2_ nanocrystals could be increased compared with that on unmodified crystals [66]. By controlling coexposed {001} and {101} facets, the highest performance was observed on TiO_2−x_{001}-{101} with a CO production rate of 55 and 27 μmol g^−1^ h^−1^ (quantum yield of 0.31 and 0.134%) under UV-VIS and visible light, respectively (Figure 6a). This excellent performance, even under visible light, can be attributed to the high CO_2_ adsorption capacity, combination of {001} and {101} facets, and the new energy state of Ti^3+^ cations, which improve the activation and conversion kinetics of the adsorbed species. Later, a facile hydrothermal method was reported to synthesize TiO_2_ with a high concentration of Ti^3+^ [67]. Because of the oxygen vacancies and coexposed {001} and {101} facets, the photocatalytic activity was improved with wide-spectrum solar light absorption to enhance CO_2_ photoconversion and CH_4_ selectivity under sunlight irradiation.

Oxygen vacancies in TiO_2_ can also be formed from the introduction of other elements to achieve high efficiencies under visible light. This is because the formation of Ti^3+^ caused by oxygen deficiency creates an intermediate band and thus accelerates electron-hole pair separation. In addition, the oxygen vacancy sites on the surface of the photocatalysts could allow adsorption of more CO_2_ compared with the bulk surface [62,68,69,70,71,72]. Ye et al. prepared an oxygen-deficient perovskite structure of self-doped SrTiO_3−δ_ for CO_2_ photoreduction under visible light [62]. Using a one-step combustion method followed by calcination in Ar at temperatures ranging from 1200 to 1400 °C, oxygen vacancies were generated coupled with Ti^3+^ to activate visible-light absorption. The increase in oxygen vacancies was found to be positively correlated with the CO_2_ chemical adsorption capacitance. The optimized SrTiO_2.83_ photocatalysts showed the highest conversion of CO_2_ in photoreduction to CH_4_ with a quantum efficiency of 0.21% at 600 nm. Analogously, for the first time, Li et al. reported the spontaneous dissolution of CO_2_ into CO even in the dark on a defective and partially oxygen-depleted Cu(I)/TiO_2−x_ surface [68]. The photocatalyst was prepared by thermal annealing of Cu(OH)_2_/TiO_2_ in an inert environment at moderate temperatures to generate surface oxygen vacancies due to partial oxygen loss and the reduction of Cu^2+^ to Cu^+^ (Figure 6b). Moreover, the oxidation state of copper on TiO_2_ induced by the pretreatment conditions can play an important role in the photoreduction performance of catalysts. The active charge separation of H_2_-pretreated Cu/TiO_2_ can be attributed to the synergistic effect of the mixture of Cu^+^ and Cu and the oxygen vacancy defects [69]. In addition, metallic Cu particles have been reported to promote the formation of oxygen vacancies in black TiO_2_-coated Cu NPs through metal-oxide interactions, thus improving the visible-light adsorption and the adsorption of CO_2_ to improve photocatalytic activity [70]. Furthermore, oxygen vacancies are also formed from the introduction of co-dopants [71]. Lee et al. reported high production yields of CH_4_ and CO under visible light, which are 933 and 588 μmol g^−1^ h^−1^, respectively, on 2Cu@4V-TiO_2_/PU (PU = polyurethane) [72].

#### 3.2.2. Surfaces of Basic Functional Sites

Because CO_2_ molecules can act as an electron acceptor because of the electrophilic carbon atom, some studies have explored the utilization of basic hydroxides, oxides, and amine functional groups to promote the chemisorption and activation of photocatalysts. Ye et al. modified the TiO_2_ surface by loading 3 wt% solid NaOH to obtain 52 μmol g^−1^ CH_4_ within 6 h; in contrast, bare TiO_2_ did not show any CH_4_-production activity [73]. Similarly, Wang et al. loaded MgO onto the TiO_2_ surface to transform CO_2_ into CO via photoreduction with H_2_O vapor [74]. The introduction of MgO into Pt-TiO_2_ can generate 2.2 μmol of CH_4_ within 10 h [75]. Lately, Li et al. coated an ultrathin MgO layer on porous TiO_2_ mixed anatase-rutile phases by atomic layer deposition (ALD) [76]. They increased the number of atomic MgO layers from 1 to 100 and found that five layers of MgO exhibited 4- and 21-fold higher CO production compared with pristine porous-TiO_2_ and common P25, respectively (Figure 6c–e). The enhanced activity can be ascribed to the increased concentration of surface Ti^3+^ species and hydroxyl groups caused by the uniform dispersion of MgO layers, which act as active sites for adsorption and photoreduction of CO_2_. Furthermore, the deposited MgO layers promoted electron-hole separation because of the passivation of TiO_2_ states on the surface. Likewise, amine-functionalized TiO_2_ was synthesized using monoethanolamine (MEA) to improve CO_2_ adsorption, thus enhancing the photocatalytic activity by approximately three times than the pristine TiO_2_ [77]. Liu et al. also reported the high visible-light harvesting ability and improved CO_2_ adsorption capacity of hierarchical amine-functionalized titanate nanosheet-based yolk@shell microspheres using one-pot diethylenetriamine mediated anhydrous alcoholysis method [78]. As a result, the conversion of CO_2_ exhibited a CH_3_OH yield of 8 μmol g^−1^ h^−1^ under visible-light irradiation.

### 3.3. Cocatalysts

The introduction of cocatalysts into TiO_2_ photocatalysts can be beneficial for CO_2_ reduction not only by enhancing the charge transfer via formation of a heterojunction, thus improving the charge carrier separation, but also by forming supplementary active sites for the reaction to increase the adsorption of CO_2_ and stabilization of intermediates in the photoreaction [79,80,81,82].

#### 3.3.1. Metal and Metal-Oxide Cocatalysts

Metals and metal oxides have been frequently employed as cocatalysts with TiO_2_ to improve the performance of TiO_2_ [35,75,81,82,83,84,85,86,87,88,89,90,91,92,93,94,95,96].

When a cocatalyst system is illuminated, a transfer of photoinduced electrons from TiO_2_ to the metallic surface possessing a larger work function at the interface can occur, and thus, metallic sites can act as an electron-sink to activate CO_2_ and generate the bent CO_2_^−^ intermediate [6,80]. Wang et al. reported the effect of noble metal cocatalysts on the photocatalytic activity of TiO_2_ and found that the rate of CH_4_ formation increased as follows: Ag < Rh < Au < Pd < Pt. This result agrees well with the increase in the charged-pair separation efficiency [75]. Pd NPs on the surface of TiO_2_ have also been reported to act as sites for the adsorption and activation of CO_2_ [82].

**Figure 7:**
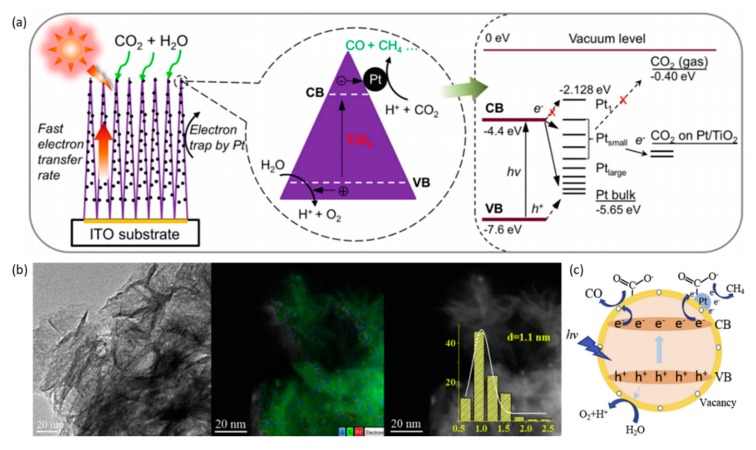
(**a**) Schematic illustration of photocatalytic reduction of CO_2_ on a Pt-TiO_2_ photocatalyst, exhibiting fast transfer of photogenerated electrons inside the highly oriented TiO_2_ single crystals into the Pt sites where the redox reaction converts CO_2_ into CH_4_. Reproduced with permission from [35]. Copyright 2017, American Chemical Society. (**b**) HRTEM images and STEM-HAADF-mapping of well-dispersed 1.1-nm Pt nanoparticles on oxygen vacancy-rich ultrathin TiO_2_. (**c**) Proposed mechanisms of the ultrathin TiO_2_-supported highly dispersed Pt nanoparticles for photoreduction of CO_2_ with H_2_O. (**b**,**c**) Reproduced with permission from [83]. Copyright 2019, Elsevier.

In addition, the size of metallic NPs is a crucial factor determining the activity and CH_4_-formation rate. The optimum size of Pt NPs on 1-D nanostructured TiO_2_ single crystals to produce CH_4_ at an excellent rate of 1361 μmol g^−1^ h^−1^ was found to be 1.04 ± 0.08 nm; the result was associated with a quantum yield of 2.41%, which is advanced compared with the production by pristine TiO_2_ and P25 catalysts [35]. The energy level of the small Pt coated on 1-D TiO_2_ was rationally positioned at a higher level than the CB edge of TiO_2_, impeding photoinduced electron transfer (Figure 7a), which is beneficial for CO_2_RR. Recently, Liu et al. synthesized ultrafine 1.1-nm Pt nanoparticles photoreduced and highly dispersed on ultrathin TiO_2_ fabricated by deprotonated ethylene glycol as a support (P-Pt/TiO_2_-U) [83]. The synergistic effects from both ultrathin TiO_2_ and ultrafine highly dispersed Pt are as follows: Increased electron rate transfer on ultrathin TiO_2_ nanosheets with abundant defects and ultra-large surface area, the facilitated separation of photogenerated electron-hole pairs induced by ultrafine Pt NPs, which was proved to improve the light-harvesting capacity and quantum efficiency, and finally, the improved adsorption ability of CO_2_ caused by the synergy of metal and support (Figure 7b,c).

As mentioned above, Cu is a popularly used transition metal for cocatalysts of TiO_2_. Numerous studies have assessed its contribution to cocatalyst systems. Cu-moieties decorated on the surface of N-doped TiO_2_ nanotubes and Cu on TiO_2_-SiO_2_ catalysts have demonstrated improved CH_4_ formation from CO_2_RR. Interestingly, Corma et al. reported the conversion of CO_2_ to formic acid with a yield of 25.7 μmol g^−1^ h^−1^ on Cu-doped TiO_2_ under UV-rich illumination with Na_2_S [84]. CuO-TiO_2_ hollow microspheres with CuO extensively dispersed on the shell have been reported to improve the light-harvesting efficiency to produce CO and CH_4_ from photoreduction of CO_2_ under UV conditions [85]. This result can be attributed to the improved electron trapping ability induced by CuO. Meanwhile, hydrogenated hollow microspheres, in which Cu^2+^ was reduced to Cu^0^, could further enhance CH_4_ production because of the hole capturing caused by Cu^0^ and H_2_ formation from water dissociation for CH_4_ evolution. Recently, Jiang and co-workers employed atomically dispersed Cu supported on ultrathin TiO_2_ nanosheets to photocatalytically reduce CO_2_ to CO [86]. Most importantly, Cu is an abundant, non-toxic, and low-cost metal, which is a potential alternative to noble metals. In this study, a method is also presented to recycle the catalyst for long-term utilization. Similarly, Cu was recently ascertained to suppress the hydrogen evolution in CO_2_ photoreduction on Cu-TiO_2_ to selectively produce CO at a high rate [81].

**Figure 8:**
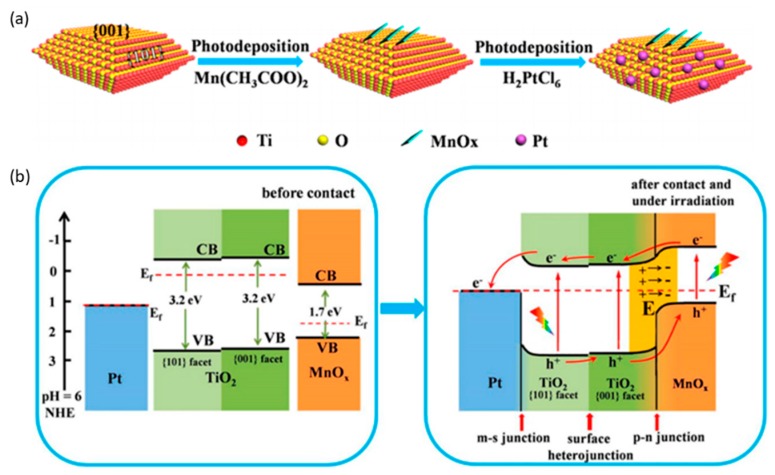
(**a**) Schematic illustrations of synthesis of hybrid TiO_2_-MnO_x_-Pt composite by photodeposition of MnO_x_ nanosheets and Pt nanoparticles on TiO_2_. (**b**) Schematic diagram of proposed photocatalytic CO_2_ reduction mechanism and modified bandgaps of the ternary TiO_2_-MnO_x_-Pt photocatalyst before contact and after contact and under irradiation. Reproduced with permission from [89]. Copyright 2018, American Chemical Society.

Furthermore, the employment of binary cocatalysts was investigated on Cu_2_O/Pt/TiO_2_ and MgO-Pt/TiO_2_ systems [75,87]. The introduction of Pt on TiO_2_ was demonstrated to promote the capture of photogenerated electrons and to hinder pair-charge recombination; however, H_2_ evolution was spontaneously increased. To suppress H_2_ generation and enhance the chemisorption and activation of CO_2_, Cu_2_O was deposited as a shell respected with Pt core, and analogously, MgO was deposited as an amorphous layer onto Pt/TiO_2_. As a result, high CH_4_ selectivities of 85% and 83% were obtained on Cu_2_O/Pt/TiO_2_ and MgO-Pt/TiO_2_ catalysts, respectively. In another study, a binary cocatalyst system of Ag/Pd was reported to enhance the photocatalytic activity for CO_2_ to CH_4_ on Ag/Pd supported on N-doped TiO_2_ with a production rate of 79 μmol g^−1^ h^−1^ [88]. Recently, a multi-heterojunction was created on TiO_2_–MnO_x_–Pt films (Figure 8). Two heterojunctions were created: Aa p–n junction between the MnO_x_ and TiO_2_ {001} facet and a metal–semiconductor junction between Pt and TiO_2_ {101} facet. Thus, efficient separation of charged pairs was obtained to produce three times higher CH_4_ and CH_3_OH compared with the pristine TiO_2_ nanosheets [89].

Another study combined some of the effective strategies mentioned above. Ye et al. fabricated a photocatalyst using hydrous hydrazine Au–Cu bimetal as the cocatalyst supported on SrTiO_3_/TiO_2_ coaxial nanoarchitecture arrays to create a heterojunction with fast electron-transfer [97]. As a result, excellent performance was reported on the catalyst with CO as the main product with a production rate of 3770 μmol g^−1^ h^−1^ along with a CH_4_ production rate of 421.1 μmol g^−1^ h^−1^. Remarkably, the production of C2+ products (C_2_H_6_: 190.1, C_2_H_4_: 73.3, C_3_H_6_: 40.8 μmol g^−1^ h^−1^, respectively) is very noticeable due to the formation of C2+ products with higher values in industry from photoreduction of the problematic CO_2_ gas. This research can be a representative study that manipulated various approaches to achieve a high production rate for photoreduction of CO_2_, shedding light on the hybrid nanostructured design for further enhancement of the rate in the future.

#### 3.3.2. Plasmonic Effect

One of the most useful effects induced by the incorporation of noble metals on the TiO_2_ surface to enhance the visible-light-harvesting efficiency and charge separation is the surface plasmon resonance (SPR). Many applications from SPR have recently emerged as an efficient approach to advance the conversion of water and CO_2_ [90,91,92]. The enhanced activity of plasmonic effect-related photocatalysts is ascribed to the direct charge transfer mechanism or the enhancement of the local electric field induced on the noble metallic atoms. Ag was loaded on TiO_2_ nanotube arrays to improve CH_4_ formation from photoreduction of CO_2_ under visible light. The improved light absorption from the nanotube arrays of TiO_2_ combined with the deposition of Ag NPs inside TiO_2_ nanotubes was believed to cause the plasmonic effect with hot electron generation [93].

**Figure 9:**
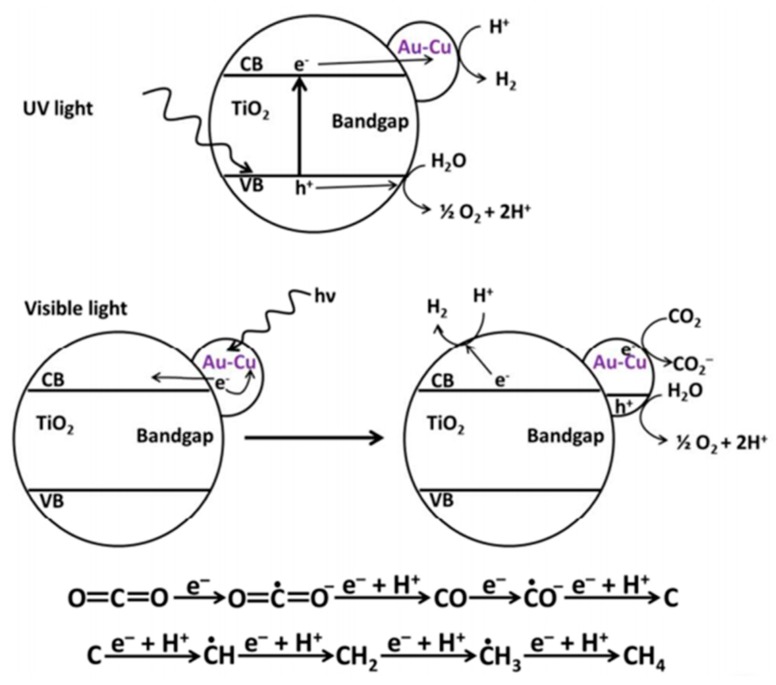
Proposed schematic illustration of the mechanism of photocatalytic CO_2_ reduction under UV and visible light for Au–Cu alloy NPs decorated on TiO_2_ as photocatalysts, demonstrating the crucial role of the irradiation wavelength range on product distribution. Reproduced with permission from [96]. Copyright 2014, American Chemical Society.

In addition, Au is also known to exhibit the SPR effect on TiO_2_. The synergistic combination of the SPR effect from Au NPs and the role of Pt as electron-sink on nanohybrid Au/Pt co-decorated on TiO_2_ nanofibers was reported for visible-light harvesting and was found to hinder charge recombination of photoexcited TiO_2_ [94]. Tahir synthesized montmorillonite (MMT) dispersed Au/TiO_2_ nanocatalysts through a simple sol-gel method [95]. The photocatalytic performance of MMT-dispersed Au/TiO_2_ under simulated solar light was enhanced because of the SPR effect of Au, catalyzing photoreduction of CO_2_ to CO with a high production rate of 1223 μmol g^−1^ h^−1^. Remarkably, Garcia et al. fabricated Au–Cu alloy nanoparticles supported on TiO_2_ to achieve excellent performance [96]. The selectivity of electrons toward CH_4_ evolution could reach 97% with a high production rate of approximately 2200 μmol g^−1^ h^−1^ under visible-light irradiation. The enhanced visible-light-harvesting ability can be ascribed to the plasmonic effect from Au. Furthermore, the presence of Cu bonding to the CO* intermediate on the photocatalyst could lead to the high selectivity of CH_4_ through a “carbene pathway” with the detection of some intermediates including CO_2_^−^, Cu-CO, and carbon deposits on the surface (Figure 9).

### 3.4. Hybrid TiO_2_ Nanocomposites

The employment of other materials into TiO_2_ photocatalysts is fruitful for CO_2_ reduction because of supplied high active sites surface area, facilitation of charge transfer to enhance CO_2_ adsorption, and impeding electron-hole recombination [98,99,100,101,102,103,104,105,106,107,108,109,110].

#### 3.4.1. Carbon-Containing Composites

Carbon-based materials possess many applicable features such as large surface areas for active sites, broad electronic properties, and various architectural nanostructures, leading to numerous applications in the previous decades. Thus, carbon materials including graphitic carbon derivatives, graphene, graphene oxide (GO), and carbon nanotubes have been incorporated with semiconductor-based materials for facial synthesis and for lowering the fabrication cost while attaining the light absorption in the visible light and enhanced charge separation and transport [98,99,100,101,102,103,104,105,106,107,108]. Liang et al. reported solvent-exfoliated graphene on GO-TiO_2_ systems to functionalize titania for the photochemical reduction of CO_2_ [99]. An increment in the visible-light harvesting was observed on the less defective solvent-exfoliated graphene caused by the fast-electrical mobility, accelerating the photoinduced electron transport into reactive sites.

Next, Zou et al. prepared a hollow spherical structure from titania Ti_0.91_O_2_ and graphene nanosheets [100]. The hollow spherical structure and the unique composition enhanced the photocatalytic activity because of the fast charge transfer, long-lasting lifetime of charge carriers, and enhancement of light absorption. In agreement with this study, carbon@TiO_2_ hollow spheres were recently reported to increase the photocatalytic activity to produce CH_4_ and CH_3_OH [101]. Importantly, the carbon content was found to be correlated to the charge transfer efficiency. In their subsequent study, Zou et al. developed an in situ reduction-hydrolysis method to synthesize TiO_2_-graphene nanosheets [102]. Using this technique, GO was reduced to graphene simultaneously with the formation of TiO_2_ from hydrolysis of Ti^4+^ dihydroxy is to create a 2D sandwich-like structure. Therein, the TiO_2_ deposited on graphene acted as a stabilizing agent for graphene nanosheets, whereas the Ti^3+^ observed on TiO_2_ NPs could trap the photoexcited electrons coupled with the suppression of the electron-hole pair recombination. Interestingly, the photocatalysts could catalyze the photoreduction of CO_2_ toward C_2_H_6_ with a production rate of 16.8 μmol g^−1^ h^−1^, which is different from other reported catalysts, proving that the architectural design of hybrid nanocomposites can vary product selectivity.

Among the carbon-based candidates, graphitic carbon nitrides (g-C_3_N_4_) with an increased concentration of nitrogen on the surface and a narrower band gap (2.7 eV) than TiO_2_ have attracted increasing attention because of the ability for CO_2_ activation. Cu-TiO_2_ was dispersed on g-C_3_N_4_ to enhance the photocatalytic performance of CO_2_ conversion to CH_4_ [103]. In another study, the charge transfer between semiconducting C_3_N_4_ nanosheets and a Ru(II)–Re(I) binuclear complex (RuRe) was improved using rutile TiO_2_ nanocrystals as a modifier [104]. The RuRe/TiO_2_/NS-C_3_N_4_ hybrid could promote both CO formation rate and turnover number under visible-light irradiation as a result of the advanced lifetime of photoinduced electrons. Zhao et al. wrapped Pt/TiO_2_-nanocrystals with reduced GO (rGO) to synthesize a core-shell structure. The surface residual hydroxyl on the rGO shell and the extended π bonds proved to increase the CO_2_ adsorption and activation, while the whole hybrid core-shell structure enhanced the electron transfer and the separation efficiency to highly selective production of CH_4_ up to 99.1% with a production rate of 41.3 μmol g^−1^ h^−1^ [105].

**Figure 10:**
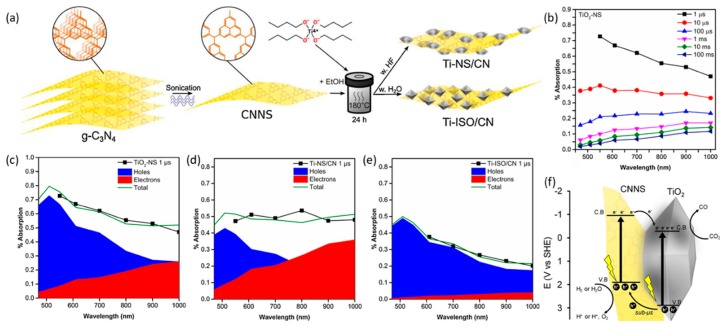
(**a**) Scheme of the synthesis procedure used to produce TiO_2_/CNNS composites. (**b**) Transient absorption spectroscopy spectra used to study the photoexcitation processes in powdered samples at different delay times of TiO_2_ nanosheets (TiO_2_-NS) following photoexcitation. Initial spectra at 1 μs for (**c**) TiO_2_-NS, (**d**) Ti-NS/CN (synthesized in the presence of HF), and (**e**) Ti-ISO/CN (synthesized in the absence of HF). The hole contribution is shaded in blue, and the electron contribution is shaded in red. The sum of the two is indicated as the total (green line) compared with the experimental values shown in the black line and squares. (**f**) Photocatalytic CO_2_ reduction reaction pathway for the TiO_2_/CNNS nanocomposites improved by hole transfer from TiO_2_-NS to CNNS. Reproduced with permission from [106]. Copyright 2019, Elsevier.

Recently, Petit et al. employed carbon nitride nanosheets (CNNS) from g-C_3_N_4_ and grew TiO_2_ and control its facets on CNNS, favoring the formation of {001} facets because of their enhanced photocatalytic activity [106]. The hybrid TiO_2_/CNNS heterostructures exhibited superior CO_2_ adsorption and charge transfer acceleration, resulting in the availability of photoexcited electrons under UV-Vis illumination, which is 10-fold higher compared with that exhibited by pristine materials. Transient absorption spectroscopy analyses showed that the hole transfer from TiO_2_ to CN was observed. The interfacial charge transfer via the heterojunction could inhibit the recombination of paired charges (Figure 10). This revealed that the fine control of TiO_2_ facets could lead to a high activity for photoreduction of CO_2_ associated with a hybrid nanocomposite structure with carbon nitride materials, thus enabling charge transfer to enhance CO_2_ adsorption, and impeding electron-hole recombination can be a promising method to increase the production rate.

Lately, Barbieri et al. reported a continuous operating system employing C_3_N_4_-TiO_2_ Nafion photocatalytic membrane reactors to examine the effects of crucial parameters including reaction pressure, H_2_O/CO_2_ feeding ratio, or contact time on the performance and selectivity of various detected products from photoreduction of CO_2_ (MeOH and HCHO) [107].

Similarly, in another work, P-O linked Z-scheme g-C_3_N_4_/TiO_2_ nanotube composites were fabricated to enhance the visible-light harvesting capability and the charge separation caused by g-C_3_N_4_ and P-O links [108]. CO_2_ photoreduction yielded 46.9 mg L^−1^ h^−1^, 38.2 mg L^−1^ h^−1^, and 28.8 mg L^−1^ h^−1^ of acetic, methanol, and formic acid, respectively, which was ~3.3, 3.5, and ~3.8 times the production from TiO_2_ nanotubes.

#### 3.4.2. Other Composites

**Figure 11:**
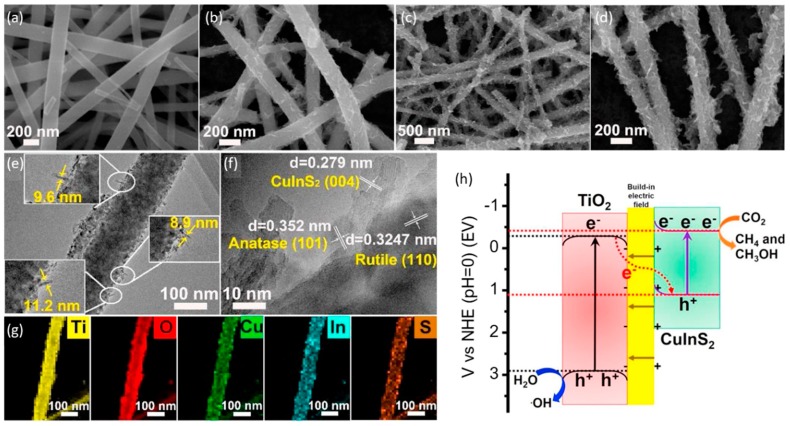
SEM images of (**a**,**b**) pristine TiO_2_, (**c**,**d**) sensitized CuInS_2_/TiO_2_. TEM image of (**e**) pristine TiO_2_ and (**f**) sensitized CuInS_2_/TiO_2_. (**g**) energy dispersive X-ray (EDX) element mapping of Ti, O, Cu, In, and S. (**h**) Scheme of the illustrated charge transfer and separation in the optimum sensitized CuInS_2_/TiO_2_ photocatalyst under simulated sunlight irradiation. Reproduced with permission from [109]. Copyright 2018, Elsevier.

Xu et al. employed an in situ hydrothermal method to deposit CuInS_2_ nanoplates onto TiO_2_ nanofibers to achieve the Z-scheme TiO_2_/CuInS_2_ heterostructure; excellent photoreduction activity of CO_2_ to CH_4_ and CH_3_OH under light irradiation was observed [109]. A mechanism was proposed for the high activity of TiO_2_/CuInS_2_ based on the direct Z-scheme heterojunction, in which photoexcited electrons from the TiO_2_ CB could be transferred and recombined with the hole of CuInS_2_ VB, whereas electrons generated on CuInS_2_ were catalyzed for the photoreduction of CO_2_ (Figure 11).

The introduction of two-dimensional inorganic compounds, MXene, as a supporter for TiO_2_ was investigated. Yu et al. grew TiO_2_ NPs on Ti_3_C_2_ in situ by calcination. An ultrathin fluffy rice crust-like structure was observed on the composite, which is beneficial for high surface for active sites. In addition, the highly conductive Ti_3_C_2_ assisted photoexcited electron transfer and prevented paired charge recombination. As a result, the TiO_2_/Ti_3_C_2_ composite achieved a 3.7 times higher CH_4_-production rate than commercial TiO_2_ (P25) [110].

## 4. Conclusions and Perspective

Photoreduction of CO_2_ is an attractive approach to utilize CO_2_ gas for producing value-added carbon chemicals to achieve multiple purposes. This method has several advantages such as contributing to the energy demands using CO_2_ as a significant carbon resource, diminishing environmental issues from CO_2_, and leveraging clean, renewable energy from sunlight. To mimic natural photosynthesis, in which solar energy is used to convert CO_2_ and H_2_O to carbohydrates and oxygen, artificial photosynthesis was performed to develop photocatalysts, which play an identical and sophisticated role like terrestrial plants to efficiently harvest sunlight and separate the photogenerated charges for reactions for the conversion of CO_2_ and H_2_O into energetic chemicals. Overall, highly efficient photoreduction of CO_2_ to fuels can be achieved by a combination of some critical strategies, including: High adsorption and activation of CO_2_, large surface areas for active sites, efficient sunlight harvesting, generation of photoexcited electron-hole pairs, accelerated charge transfer to reactive site, elongated lifetime of charge carriers, and effective inhibition of charge recombination. The present review reveals the significant recent progress on TiO_2_-based photocatalysts and decisive factors for photocatalytic performance via some critical strategies in Table 2.

The architectural structure design of photocatalysts, including crystal phases, oriented facets, and dispersion of TiO_2_ active sites, can play a crucial role in driving the CO_2_ reduction pathways and improving photocatalytic activity. Recently, tremendous achievements have been reported with respect to the influential structure of TiO_2_ rutile and anatase phases, morphology, size, various facets including {101}, {001}, {110}, and {111}, and alternative methods for TiO_2_ dispersion on porous materials to improve their intrinsic photoactivities. Moreover, the introduction of oxygen defects/vacancies and functional basic sites into the TiO_2_ surface can modify intermediate affinity and significantly improve CO_2_ adsorption and activation ability for CO_2_ reduction. Importantly, the utilization of cocatalysts such as noble metals, transition metals, and their oxides via plasmonic effects or/and heterojunctions improves visible-light harvesting, increases charge separation efficiency, and impedes electron-hole recombination. Furthermore, TiO_2_-based hybrid nanocomposites with advanced performances have emerged recently by manipulating several efficient approaches mentioned above combined with the hybridization with other materials (e.g., graphene, GO, rGO, g-C_3_N_4_ and its derivatives, sensitized-materials, and 2D MXene) to overcome the limitations of Ti and TiO_2_-based materials, exhibiting a CO_2_ photoreduction production rate of some thousands of micromoles per gram per hour.

Nonetheless, further improvement of CO_2_ adsorption, activation, and visible-light harvesting needs to be examined to enhance the sunlight harvesting yield and rate of production, which is, however, still far from practical applications. Insights on the reaction mechanism should also be investigated via in situ measurements with several powerful techniques for understanding and controlling reaction pathways, resulting in high selectivity of major products. In addition, most of studies reported the popular distribution of C1 products (CH_4_, HCOOH, CH_3_OH), especially CH_4_, from photoreduction of CO_2_ while there is a lack of the formation of C2+ products with higher price and demand in industry although a few studies exhibited the formation of C_2_H_4_, C_2_H_6_, and C_3_H_8_ as mentioned above (see Table 2). However, the production of C2+ products is much more complex, which requires a deeper understanding of mechanism and control. Furthermore, mass transfer and charge transfer should be comprehensively considered by examining the influence of operating parameters, structure of photocatalysts, and reactor design and operation to enable advanced yield and efficiency of sustainable CO_2_ photoreduction.

## Figures and Tables

**Table 1:** Products, number of transferred electrons, and standard potentials vs. normal hydrogen electrode (NHE) in reduction of CO_2_ at pH = 7.

Product	Reaction	*n*	E^o^ (V vs. NHE)
Carbon monoxide	CO_2_ + 2H^+^ + 2e^−^→CO + H_2_O	2	−0.51
Formic acid	CO_2_ + 2H^+^ + 2e^−^→HCOOH	2	−0.58
Oxalate	2CO_2_ + 2H^+^ + 2e^−^→H_2_C_2_O_4_	2	−0.87
Methanol	CO_2_ + 6H^+^ + 6e^−^→CH_3_OH + H_2_O	6	−0.39
Methane	CO_2_ + 8H^+^ + 8e^−^→CH_4_ + 2H_2_O	8	−0.24
Ethanol	2CO_2_ + 12H^+^ + 12e^−^→C_2_H_5_OH + 3H_2_O	12	−0.33
Ethane	2CO_2_ + 14H^+^ + 14e^−^→C_2_H_6_ + 4H_2_O	14	−0.27
Hydrogen	H_2_O + 2e^−^→2OH^−^ + H_2_	2	−0.41

**Table 2:** Summary of photocatalytic CO_2_ reduction performance of various photocatalysts.

Photocatalysts	Reaction Conditions	Production Rate (μmol g^−1^ h^−1^)	Reference (Year)
Anatase TiO_2_ by coexposed {001} and {101} facets	300 W Xe lamp TiO_2_-NaHCO_3_-HCl (solid-liquid)	CH_4_: 1.35	[52] (2014)
Cubic anatase TiO_2_ nanocrystals (100 ± 13 nm)	300 W Xe lamp TiO_2_-H_2_O-CO_2_ (solid-liquid)	CH_4_: 4.56 CH_3_OH: 1.48	[53] (2015)
TiO_2_ nanosheets with exposed {001} facet	2 × 18 W Hg lamps TiO_2_-H_2_O-CO_2_-NaOH (solid-liquid)	CH_4_: 0.2 CO: 0.12 CH_3_OH: 0.19 HCHO: 0.066	[54] (2014)
Flame-annealed TiO_2_ nanotubes formed in aqueous electrolyte	AM 1.5G, 100 mW cm^−2^ Catalysts-H_2_O-CO_2_ (solid-gas)	CH_4_: 156.5	[56] (2019)
Nanotubes/nanowires assembled from TiO_2_ nanoflakes with {111} facet	300-W Xe lamp TiO_2_-H_2_O-CO_2_-(CH_3_)_2_CHOH (solid-liquid)	CH_4_: 1708.1 CO: 463.2	[57] (2019)
Highly dispersed titanium oxide on mesoporous SBA-15 (Ti-SBA-15)	100-W Hg lamp (>250 nm) TiO_2_-H_2_O-CO_2_ (solid-gas)	CH_4_: 106 CH_3_OH: 27.7	[61] (2005)
Oxygen-deficient blue TiO_2_ nanocrystals with coexposed {101} and {001} facets	100-W Hg lamp 450-W Xe lamp TiO_2_-H_2_O-CO_2_ (solid-gas)	CO: 55 (UV-VIS) CO: 27 (Visible light)	[66] (2016)
Cu and V co-doped TiO_2_ (P25) deposited on polyurethane Cu@V/TiO_2_-PU	Visible light (2 × 20 W white bulbs) Catalysts-H_2_O-CO_2_ (solid-gas)	CH_4_: 933 CO: 588	[72] (2017)
3% NaOH-surface modification TiO_2_ (ST01)	300-W Xe lamp Catalysts-H_2_O-CO_2_ (solid-gas)	CH_4_: 8.7	[73] (2014)
5 ultrathin MgO layers deposited on porous TiO_2_ mixed anatase-rutile phases by atomic layer deposition	450-W Xe lamp Catalysts-H_2_O-CO_2_ (solid-gas)	CO: 54	[76] (2019)
TiO_2_-0.5% Ag	100-W Xe lamp (320–780 nm) Catalysts-H_2_O-CO_2_ (solid-gas)	CH_4_: 2.1	[75] (2014)
TiO_2_-0.5% Au	100-W Xe lamp (320–780 nm) Catalysts-H_2_O-CO_2_ (solid-gas)	CH_4_: 3.1	[75] (2014)
TiO_2_-0.5% Rh	100-W Xe lamp (320–780 nm) Catalysts-H_2_O-CO_2_ (solid-gas)	CH_4_: 3.5	[75] (2014)
TiO_2_-0.5% Pd	100-W Xe lamp (320–780 nm) Catalysts-H_2_O-CO_2_ (solid-gas)	CH_4_: 4.3	[75] (2014)
TiO_2_-0.5% Pt	100-W Xe lamp (320–780 nm) Catalysts-H_2_O-CO_2_ (solid-gas)	CH_4_: 4.3	[75] (2014)
1-D nanostructured TiO_2_ single crystals loaded with Pt nanoparticles	400-W Xe lamp Catalysts-H_2_O-CO_2_ (solid-gas)	CH_4_: 1361 CO: 200	[35] (2012)
Ag-Pd on N-doped TiO_2_ NSs	300-W Xe lamp Simulated sunlight Catalysts-H_2_O-CO_2_ (solid-liquid)	CH_4_: 79	[88] (2018)
Montmorillonite (MMT) dispersed Au/TiO_2_ nanocatalyst	Simulated sunlight Catalysts-H_2_O-CO_2_ (solid-gas)	CO: 1223	[95] (2017)
TiO_2_ powder (Degussa P25) loaded with Au–Cu alloy nanoparticles	1000-W Xe lamp Catalysts-H_2_O-CO_2_ (solid-gas)	CH_4_: 2200	[96] (2014)
Au–Cu bimetal as cocatalyst supported on SrTiO_3_/TiO_2_	300-W Xe lamp Catalysts-H_2_O-CO_2_ (solid-gas)	CO: 3770 CH_4_: 421.2 C_2_H_6_: 190.1 C_2_H_4_: 73.3 C_3_H_6_: 40.8	[97] (2015)
TiO_2_-graphene 2D sandwich-like hybrid nanosheets	500-W Xe lamp Catalysts-H_2_O-CO_2_ (solid-gas)	C_2_H_6_: 16.8 CH_4_: 8	[102] (2013)
2.5% CuInS_2_/TiO_2_	350-W Xe lamp Catalysts-H_2_O-CO_2_ (solid-gas)	CH_4_: 2.5 CH_3_OH: 0.86	[109] (2018)
TiO_2_/carbon nitride nanosheet nanocomposites	300-W Xe lamp (>325 nm) Catalysts-H_2_O-CO_2_ (solid-gas)	CO: 1.96	[106] (2019)
